# Formulation of Bicelles Based on Lecithin-Nonionic Surfactant Mixtures

**DOI:** 10.3390/ma13143066

**Published:** 2020-07-09

**Authors:** Kenji Aramaki, Keita Adachi, Miho Maeda, Jitendra Mata, Junko Kamimoto-Kuroki, Daisuke Tsukamoto, Yoshikazu Konno

**Affiliations:** 1 Graduate School of Environment and Information Sciences, Yokohama National University, Yokohama 240-8501, Japan; adachi-keita-wt@ynu.jp (K.A.); maeda-miho-wf@ynu.jp (M.M.); 2 Australian Centre for Neutron Scattering, Australian Nuclear Science and Technology Organisation (ANSTO), Lucas Heights, New South Wales 2234, Australia; jtm@ansto.gov.au; 3 Research and Development Division, KOSÉ Corporation, Tokyo 114-0005, Japan; j-kamimoto@kose.co.jp (J.K.-K.); daisuke-tsukamoto@kose.co.jp (D.T.); y-konno@kose.co.jp (Y.K.)

**Keywords:** bicelle, temperature robustness, lecithin, phospholipid, nonionic surfactant, dynamic light scattering, small-angle neutron scattering, self-assembly, nano colloid

## Abstract

Bicelles have been intensively studied for use as drug delivery carriers and in biological studies, but their preparation with low-cost materials and via a simple process would allow their use for other purposes as well. Herein, bicelles were prepared through a semi-spontaneous method using a mixture of hydrogenated soybean lecithin (SL) and a nonionic surfactant, polyoxyethylene cholesteryl ether (ChEO_10_), and then we investigated the effect of composition and temperature on the structure of bicelles, which is important to design tailored systems. As the fraction of ChEO_10_ (*X*_C_) was increased, a bimodal particle size distribution with a small particle size of several tens of nanometers and a large particle size of several hundred nanometers was obtained, and only small particles were observed when *X*_C_ ≥ 0.6, suggesting the formation of significant structure transition (liposomes to bicelles). The small-angle neutron scattering (SANS) spectrum for these particles fitted a core-shell bicelle model, providing further evidence of bicelle formation. A transition from a monomodal to a bimodal size distribution occurred as the temperature was increased, with this transition taking place at lower temperatures when higher SL-ChEO_10_ concentrations were used. SANS showed that this temperature-dependent size change was reversible, suggesting the SL-ChEO_10_ bicelles were stable against temperature, hence making them suitable for several applications.

## 1. Introduction

Liposomes are spherical molecular assemblies consisting of phospholipid bilayers. There are a wide variety of applications in pharmaceutical, biological, and cosmetic fields [1,2]. The advantages of liposomes are their encapsulation ability and controlled release of both water- and oil-soluble active ingredients [3]. Liposomes are particularly suitable as transdermal drug delivery systems (TDDS) because of their enhanced drug delivery efficiency and high skin penetration ability, both of which are due to the similarities between the lamellar structures of liposomes and the intercellular lipid layers [4]. A bicelle is another form of bilayer nanoparticle, which is typically a lipid nanodisc formed by mixtures of long-chain and short-chain phospholipids [5,6,7]. A bicelle has a flat bilayer disk with a rim, which is mainly formed by long-chain phospholipids and hydrophilic cosurfactants, respectively [8]. Alternative formulations of bicelles are also possible by mixtures of phospholipids and hydrophilic cosurfactants such as cholesterol sulfate [9], dodecyltrimethylammonium chloride (DTAC) [10], and polyoxyethylene sorbitan monooleate (Tween 80) [11,12]. Nonionic surfactants, such as Tween 80, which has much lower critical micelle concentrations than short-chain phospholipids or other ionic cosurfactants, are beneficial for their resistance to dilution as well as for efficient bicelle formulation. In addition, the combination of phospholipids and a nonionic surfactant provides denser packing of hydrophobic chains because of moderate repulsions between head groups, which can improve stability of the bicelles. A variety of applications have been reported for bicelles, including cell membrane models [13], membrane protein crystallization [14], protein structure studies [15], drug delivery carriers [16,17,18], coating materials [19], and nanomaterial synthesis [20]. One can control bicelle size by varying the mixing composition of lipid mixtures [21]. As for the TDDS, bicelles can penetrate deeper into the narrow intercellular spaces of the stratum corneum (SC) than liposomes because of their smaller size [22,23].

Most fundamental studies of bicelles, especially those for drug delivery systems, have employed phospholipids with high purity, while bicelles using low-cost materials are demanded for in various applications such as cosmetics and household products. It is also necessary to avoid complicated formulation processes such as thin-film formation. Wu et al. reported a semi-spontaneous method of vesicle preparation, in which materials were dissolved in a liquid alcohol and then mixed with water using a homogenizer [24]. In our previous report, we applied this method to a bicelle formulation based on soybean lecithin and Tween 80 [11]. In this report, we present that the lecithin-nonionic surfactant bicelles, prepared by using low-cost materials and by means of the semi-spontaneous method, have better temperature robustness compared to the typical bicelles [25]. In addition, the effects of temperature and concentration were studied, as these are known to influence the structural stability of bicelles [25]. Structural characterization was mainly performed by small-angle neutron scattering (SANS) as well as dynamic light scattering (DLS). SANS is a powerful tool to characterize bicelle structures [23] because of the possibility to obtain structural information on a wide range of length scales (approximately 1–500 nm) as well as the inner structures of bicelles.

## 2. Materials and Methods

Hydrogenated soybean lecithin (SL) was obtained from YMC Ingrid Co. (Kobe, Japan). Poly(oxyethylene) cholesteryl ethers (ChEO_10_) were obtained from Nihon Emulsions Co. (Tokyo, Japan) under the product code CS-10, and contained an average of ten ethyleneoxide units. Dipropyleneglycol (DPG) was obtained from Wako Pure Chemical Co. (Osaka, Japan). Water was filtered using a reverse osmosis system (Elix3, Millipore, Burlington, MA, USA) before use as a solvent for preparing samples. Deuterium oxide (D_2_O) (provided by Australian Nuclear Science and Technology Organisation, Lucas Heights, Australia) was used instead of light water for small-angle neutron scattering (SANS) measurements.

SL and ChEO_10_ were dissolved in DPG with the weight ratio (SL + ChEO_10_)/DPG = 1/10, followed by the addition of water until the water concentration reached 78 wt%. Sample compositions in the latter sections are described by the following:

*X*_C_: ChEO_10_ weight fraction in the SL-ChEO_10_ mixture
*W*_S_: Weight fraction of the SL-ChEO_10_ mixture in the system

An ultrasonication apparatus (Smurt, Microtech Nichion Co., Funabashi, Japan) operated at 50 W and 20 kHz was used for sample preparation. After sonication was applied for 5 min, the samples were kept in a water bath at 25 °C.

DLS measurements were performed with HPPS (Malvern, Malvern, UK) to obtain intensity-based particle size distributions. A 3.0 mW He-Ne laser (λ = 633 nm) was used as a light source. Sample temperature was controlled by using a Peltier unit within the HPPS.

SANS measurements were performed using the QUOKKA SANS instrument (Australian Nuclear Science and Technology Organisation, Lucas Heights, Australia) at the Open Pool Australian Lightwater (OPAL) reactor of the Australian Neutron Science and Technology Organization (ANSTO). Details of this instrument have been described in literature [26]. Wavelengths of 5.0 and 8.1 Å at 10% wavelength resolution were used with sample aperture diameters of 10 mm. Three instrument configurations, namely source-to-sample and sample-to-detector distances of 20 and 20 m (with lens optics); 12 and 12 m; and 12 and 1.3 m, respectively, were used. A QUOKKA macro in Igor Pro software (Wavemetrics, Lake Oswego, OR, USA) originally written by Kline [27] was used. Merging of the three data sets per sample gave a continuous *q*-range between 0.0074 and 0.738 Å^−1^. Samples were measured at various temperatures in quartz cells with a path length of 2 mm (Hellma GmbH & Co., Mullheim, Germany). Curve fitting to the core-shell bicelle model [28] was performed using SasView small-angle scattering software [29].

## 3. Results

### 3.1. Particle Size in the Aqueous SL-ChEO_10_ System

**Figure 1:**
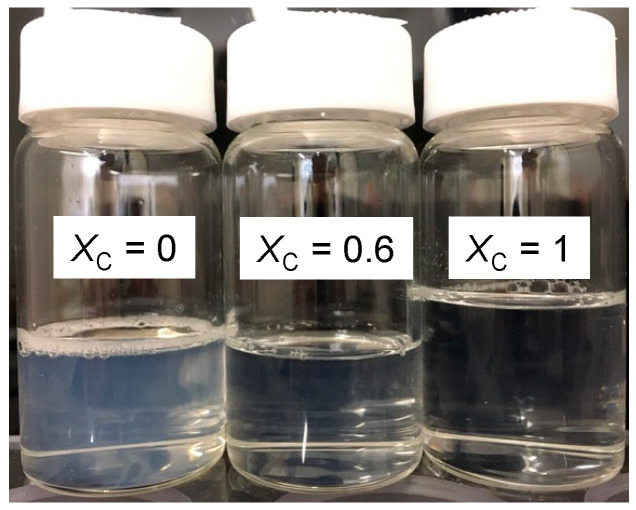
Appearance of soybean lecithin (SL)-ChEO_10_ samples (*W*_S_ = 0.02) at different *X*_C_.

We prepared samples containing SL-ChEO_10_ mixtures (*W*_S_ = 0.02) at various *X*_C_. The appearance of the samples changed according to *X*_C_, as shown in Figure 1. At *X*_C_ = 0, a transparent bluish solution was seen, which was a liposome dispersion. At *X*_C_ = 0.6, the turbidity decreased, and a transparent and colorless solution was obtained at *X*_C_ = 1.

**Figure 2:**
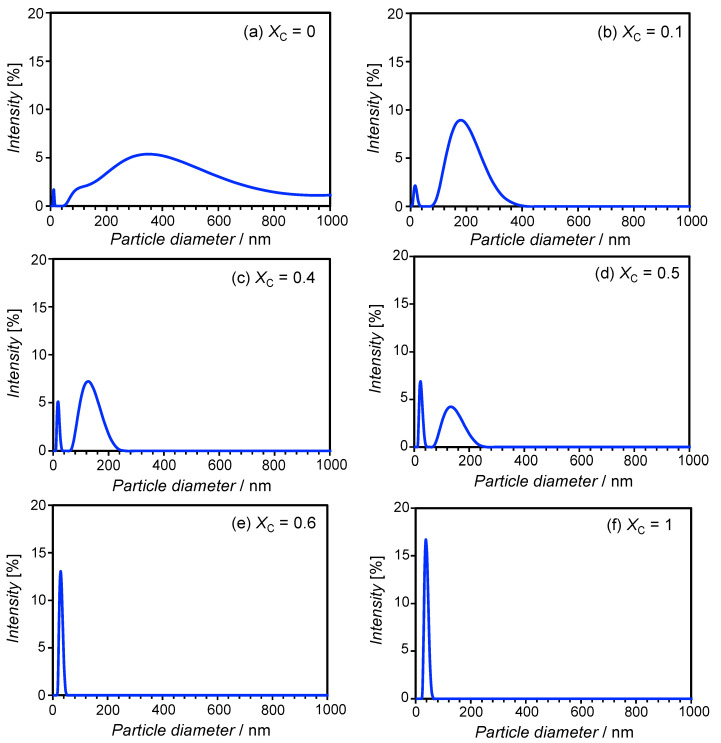
Intensity-based particle size distributions of dispersions of the SL-ChEO_10_ system (*W*_S_ = 0.02) at different *X*_C_ at 25 °C. (**a**) *X*_C_ = 0; (**b**) *X*_C_ = 0.1; (**c**) *X*_C_ = 0.4; (**d**) *X*_C_ = 0.5; (**e**) *X*_C_ = 0.6; (**f**) *X*_C_ = 1.

Figure 2 shows the DLS results obtained at various *X*_C_. At *X*_C_ = 0 (Figure 2a), a broad size distribution ranging from 100–1000 nm was obtained, which is the typical size of multilamellar-type phospholipid liposomes. On the other hand, the system containing the surfactant ChEO_10_ alone (*X*_C_ = 1, Figure 2f) resulted in a narrow size distribution that peaked at around 40 nm. Sato et al. reported the behavior of ChEO_10_ in the aqueous phase, showing that the micellar phase existed at concentrations of up to 30 wt% at 25 °C, while a 2D-rectangular (ribbon) phase formed at higher concentrations [30]. Danino et al. [31] reported that ChEO_10_ micelles formed at 0.5 wt% were disc-like with a diameter of approximately 13 nm and a thickness of approximately 4 nm, which was confirmed by cryo-TEM, with a disc-to-ribbon transition occurring as ChEO_10_ concentration was increased. Their work found slightly elongated disc or short ribbon shapes at 2 wt% ChEO_10_, indicating that the diameters obtained in the present study (~40 nm) likely demonstrate the presence of such short ribbon micelles. The mixed SL-ChEO_10_ system at *X*_C_ = 0.1 (Figure 2b) showed a bimodal size distribution, with a sharp distribution at around 20 nm and a broad distribution ranging from 80–400 nm. Bimodal distributions were also seen at *X*_C_ = 0.4 and 0.5 (Figure 2c,d); however, the broad peak shifted toward the smaller one, and the height of the sharp peak increased with ChEO_10_ content. At *X*_C_ = 0.6 (Figure 2e), only a sharp size distribution remained at around 30 nm.

During the sample preparation process, the *X*_C_ = 1 sample appeared transparent even before ultrasonication, suggesting spontaneous formation of micelles. On the other hand, the *X*_C_ = 0.6 sample was bluish and translucent before ultrasonication, indicating the presence of particles with sizes of several hundred nanometers. Therefore, the particles at *X*_C_ = 0.6 were not formed spontaneously after ultrasonication, indicating that these particles were bicelles despite the similarities between the size distributions at *X*_C_ = 0.6 and *X*_C_ = 1. The bimodal distributions seen at *X*_C_ = 0.1–0.5 indicate the coexistence of liposomes and bicelles. Such coexistence has also been reported in the DMPC-DTAC system [10] and in the egg lecithin-Tween 80 system [12].

### 3.2. Characterization of Bicelles by SANS

**Figure 3:**
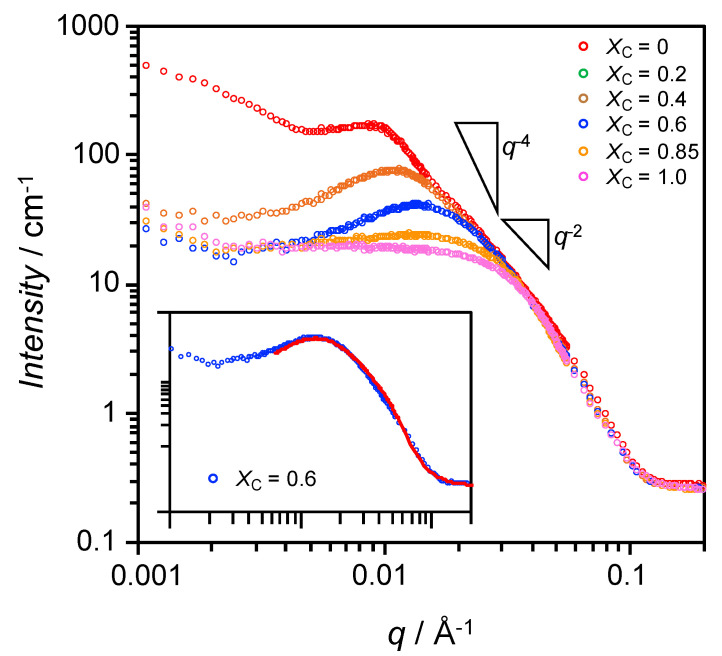
Small-angle neutron scattering (SANS) results at 25 °C for dispersions of the SL-ChEO_10_ system (*W*_S_ = 0.02) at different mixing fractions, *X*_C_. Inset shows a best fit (solid line) of the core-shell bicelle model to the scattering curve for *X*_C_ = 0.6 (fitting parameters are shown in Table S1).

SANS can be used to characterize the precise structures of bicelles, [25,32,33] allowing information regarding their inner structures to be obtained. To confirm the formation of bicelles at *X*_C_ = 0.6, SANS measurements were performed at 25 °C for samples at various *X*_C_, and the results are shown in Figure 3. The pure SL sample (*X*_C_ = 0) showed a decay of intensity with a slope of −2 on the log-log plot, which is characteristic of liposomes [34]. The strong increase in scattering intensity, *I*(*q*), at low *q* indicates the presence of large particles, since the scattering intensity at *q* = 0 is dependent on the size of the particles. This reflects the presence of liposomes with sizes of several hundred nanometers, as shown in Figure 2a. The shoulder at around *q* = 0.01 Å^−1^ (length scale ~63 nm) indicates strong interactions between particles. At *X*_C_ = 0.2, the low-*q* intensity had significantly decreased, and it gradually decreased further at higher *X*_C_. At *X*_C_ = 0.6, the scattering curve had a decay of between *q*^−2^ and *q*^−4^, suggesting bicelle formation [25]. At values above *q* = 0.03 Å^−1^, the scattering curves were almost independent of the SL-ChEO_10_ mixing fraction.

**Figure 4:**
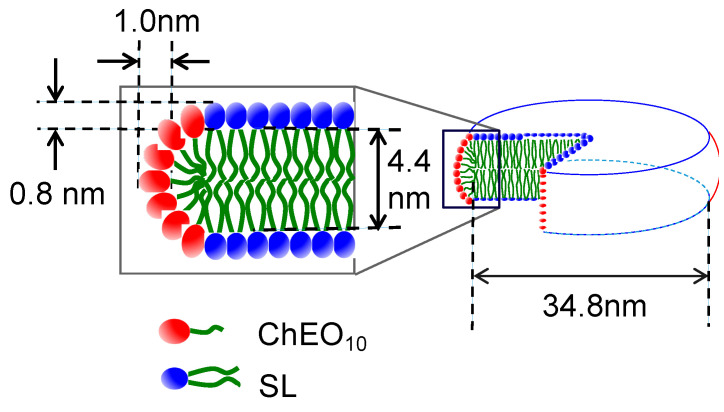
Structure of a bicelle in the SL-ChEO_10_ system (*W*_S_ = 0.02) at *X*_C_ = 0.6 and at 25 °C.

As shown in the inset of Figure 3, the scattering curve at *X*_C_ = 0.6 was successfully fitted by using the core-shell bicelle form factor model [28] and the Hayter-MSA structure factor model [35,36]. The representative structural parameters are shown in Figure 4. The disk diameter is larger while the thickness is smaller than the bicelles in the SL-Tween 80 system [11]. The maximum linear hydrocarbon chain length is described by Tanford [37] as (0.154 + 0.1265 *n*) nm, where *n* is the carbon number. The hydrophobic chains of SL are mainly C16 or C18, which give maximum lengths of 2.18 and 2.43 nm, respectively. The bilayer core thickness obtained was 4.4 nm, which is a reasonable value for twice the lengths of the C16 or C18 chains after allowing for slight shrinkage or interdigitation of the chains within the bilayer. Additionally, this indicates that the disc part of the bicelle was primarily composed of SL, and therefore that ChEO_10_ molecules were segregated to the rim. Based on this consideration, we can estimate the number of SL molecules per bicelle as follows: the area of the disc part of a bicelle (the blue circle in Figure 4) is obtained from a disc diameter of 34.8 nm is 950.7 nm^2^. The number of phospholipid molecules can then be estimated by dividing the total area of upper and lower surfaces of the disc part by the area occupied by phosphatidylcholine at the interface (0.711 nm^2^ [38]), giving a value of 2674 molecules. A slightly larger hydrophilic shell thickness at the rim than at the disc part would be reasonable if we consider longer head group ChEO_10_ molecules than SL.

### 3.3. Effect of Temperature and Concentration on Structure of Bicelles

**Figure 5:**
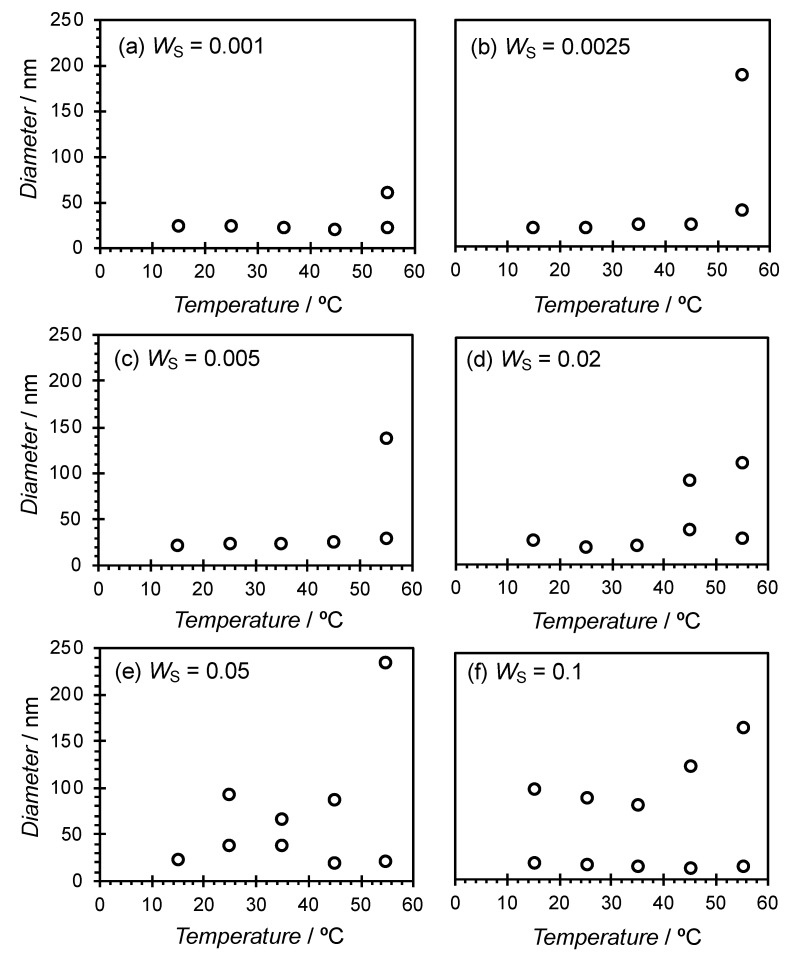
Temperature dependence of particle size when *W*_S_ = (**a**) 0.001, (**b**) 0.0025, (**c**) 0.005, (**d**) 0.02, (**e**) 0.05, and (**f**) 0.1.

The particle diameter distributions for the samples at *X*_C_ = 0.6 and *W*_S_ = 0.005 at different temperatures as obtained by DLS measurements are shown in Figure S1. Only particles with relatively small distributions were observed at temperatures up to 45 °C, while bimodal distributions with larger particle size distributions were observed at 55 °C. Particle diameters were also plotted against temperature at different *W*_S_ (Figure 5). At *W*_S_ = 0.001–0.005, the distributions were monomodal at temperatures of up to 45 °C and bimodal at 55 °C. However, when *W*_S_ was 0.02 or higher, the temperature at which the transition occurred from a monomodal to a bimodal distribution declined as *W*_S_ increased. In addition, the size of the larger particles increased with temperature.

**Figure 6:**
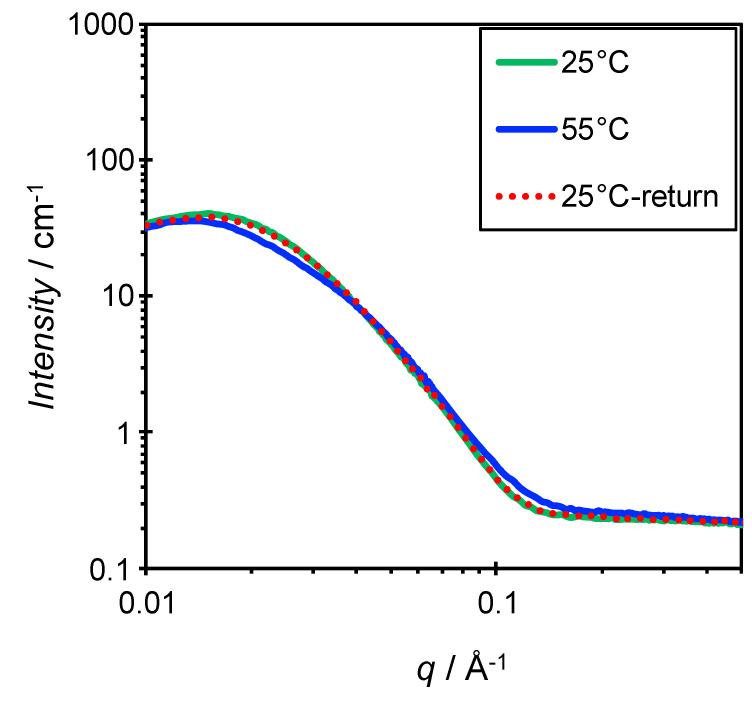
SANS results at 25 °C and 55 °C for dispersions of the SL-ChEO_10_ system (*X*_C_ = 0.6, *W*_S_ = 0.02). The “25 °C-return” indicates the measurement taken at 25 °C after cooling from 55 °C.

SANS measurements were performed at 15–55 °C for a sample where *X*_C_ = 0.6 and *W*_S_ = 0.02. The scattering curves were almost identical from 15–35 °C, as shown in Figure S2, indicating that the shape, size, and interactions of the aggregates were unchanged. This is consistent with the absence of change in particle size in this temperature range according to the DLS measurements. At 45 and 55 °C, the intensity at around *q* = 0.02–0.03 Å^−1^ decreased slightly. After finishing the measurement at 55 °C, the sample was cooled to 25 °C and another SANS measurement was taken, with identical scattering curves being obtained at 25 °C before and after heating (Figure 6).

## 4. Discussion

**Figure 7:**
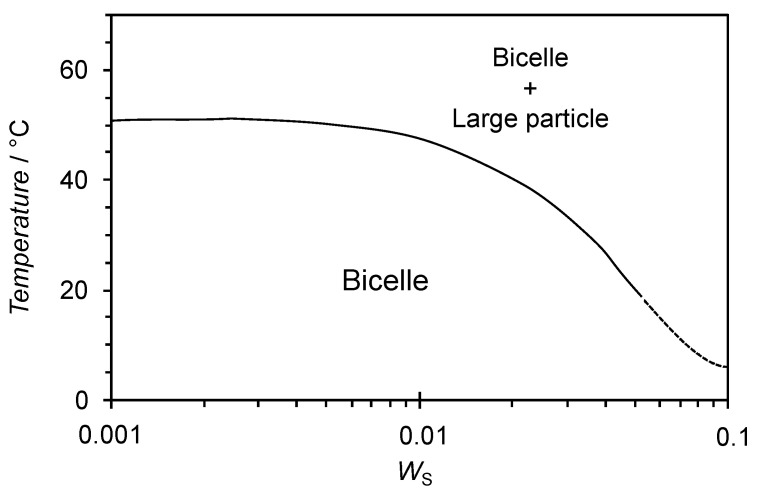
Aggregate map in the SL-ChEO_10_ system (*X*_C_ = 0.6).

As shown in Figure 5, the increases in *W*_S_ and temperature resulted in the formation of large aggregates that differed from the bicelle. Phase behavior as a function of composition and temperature is presented in Figure 7. A similar aggregate map has been reported in the dimyristoyl phosphatidylcholine (DMPC)-3-[(3-cholamidopropyl)dimethylammonio]-2-hydroxy-1-propanesulfonate (CHAPSO) system [25], which shows the formation of ribbon structures and liposomes in addition to the bicelle. On the other hand, the present system shows much simpler phase behavior. Focusing on the effects of temperature and concentration on bicelle formation in the present system, large aggregates are formed by increasing both parameters. There are two possible explanations for the observation of large aggregates at high *W*_S_ and temperatures. The first is structural transitions to different aggregated forms. The poly(oxyethylene) chain of ChEO_10_ dehydrates with both concentration and temperature, and hence the critical packing parameter of the molecule increases as the head area decreases. As a result, ChEO_10_ cannot form the rim of the bicelle, and the shape of the bicelle cannot be maintained. For example, transitions of bicelles into vesicles or ribbon structures have been reported in the literature [25]. The second explanation is the formation of clusters of bicelles that maintain their shape. In the former case, a large change in the SANS profile would be seen, but this was not observed in the result obtained at a high temperature, 55 °C, in Figure 6. In addition, temperature reversibility was seen in the SANS profile. Since the change in the SANS profile was small even at 55 °C, it is assumed that the shape of the bicelle was maintained throughout the measured temperature range, and that the large particles observed by the DLS measurements at high temperatures were clusters of bicelles. This observation shows the robustness of the SL-ChEO_10_ bicelles against temperature changes. It has been known that the gel-liquid crystal phase transition is disappeared by mixing cholesterol in phospholipid or surfactant membranes due to the phase transition to the liquid ordered (L_O_) phase, which is an intermediate phase between the liquid disordered (liquid crystalline) phase and the gel (crystalline) phase [39,40]. This results in the improved stability of liposomes or vesicles against temperature history since the L_O_ phase has no phase transition in a wide temperature range. The ChEO_10_ used in the present system can also be mixed in the bilayer membrane of bicelles, not only in the rim of bicelles. Since the ChEO_10_ has a cholesteryl group in the hydrophobic part, the mixing of ChEO_10_ in the bilayer could induce the phase transition to the L_O_ phase, hence the improved temperature robustness in the present system compared to the general bicellar systems composed of lipids only with hydrocarbon chains. It is known that the L_O_ phase formation or the improvement of low-temperature molecular alignment in bicelles occurs by mixing cholesterol sulphate (CS) in phospholipid bicellar systems [9,41]. However, further studies are necessary for the confirmation of the phase transition in the present system to understand the mechanisms fully. The formation of the clustered bicelles was not convincing at high concentrations because we could not perform the SANS measurements, which also need further studies.

## 5. Conclusions

Disc-shaped bilayer dispersions known as bicelles were prepared by mixing hydrogenated soybean lecithin (SL) and poly(oxyethylene) cholesteryl ether (ChEO_10_) via the semi-spontaneous method using a solvent and an ultrasonicator. Dynamic light scattering and small-angle neutron scattering measurements showed that the aggregates changed structure from liposomes to bicelles as SL was replaced with ChEO_10_. The results presented herein demonstrate that bicelles can be obtained with low-cost materials and a simple preparation method. In addition, the SL-ChEO_10_ bicelles were found to reversibly form aggregates when the temperature was increased, but no transitions to other aggregated structures were observed. Therefore, the SL-ChEO_10_ bicelles were found to be stable against temperature. The temperature robustness should contribute to long-term storage stability of bicellar products.

## Supplementary Materials

The following are available online at https://www.mdpi.com/1996-1944/13/14/3066/s1, Figure S1: Particle diameter distributions of *W*_S_ = 0.005 and *X*_C_ = 0.6 at (a) 15 °C, (b) 25 °C, (c) 35 °C, (d) 45 °C, (e) 55 °C, Figure S2: SANS results at different temperatures for dispersions of the SL-ChEO_10_ system (*X*_C_ = 0.6, *W*_S_ = 0.02), Table S1: SANS fitting parameter.
