# Supplementary material for: Formulation of Bicelles Based on Lecithin-Nonionic Surfactant Mixtures
Source: Materials (Basel). 2020 Jul 9;13(14):3066. doi: 10.3390/ma13143066 (PMC7412056; doi:10.3390/ma13143066)
Supplement: Supplementary file 1 [file materials-13-03066-s001.pdf]

materials

Supplementary Materials

Formulation of Bicelles Based on Lecithin-Nonionic Surfactant Mixtures

Kenji Aramaki [1,*], Keita Adachi [1], Miho Maeda [1], Jitendra Mata [2], Junko Kamimoto-Kuroki [3], Daisuke Tsukamoto [3] and Yoshikazu Konno [3]

1 Graduate School of Environment and Information Sciences, Yokohama National University, Yokohama 240-8501, Japan.; adachi-keita-wt@ynu.jp (K.A.); maeda-miho-wf@ynu.jp (M.M.)
2 Australian Centre for Neutron Scattering, Australian Nuclear Science and Technology Organisation (ANSTO), Lucas Heights, New South Wales 2234, Australia; jtm@ansto.gov.au
3 Research and Development Division, KOSÉ Corporation, Tokyo 114-0005, Japan; j-kamimoto@kose.co.jp (J.K.-K.); daisuke-tsukamoto@kose.co.jp (D.T.); y-konno@kose.co.jp (Y.K.)
* Correspondence: aramaki-kenji-cr@ynu.ac.jp

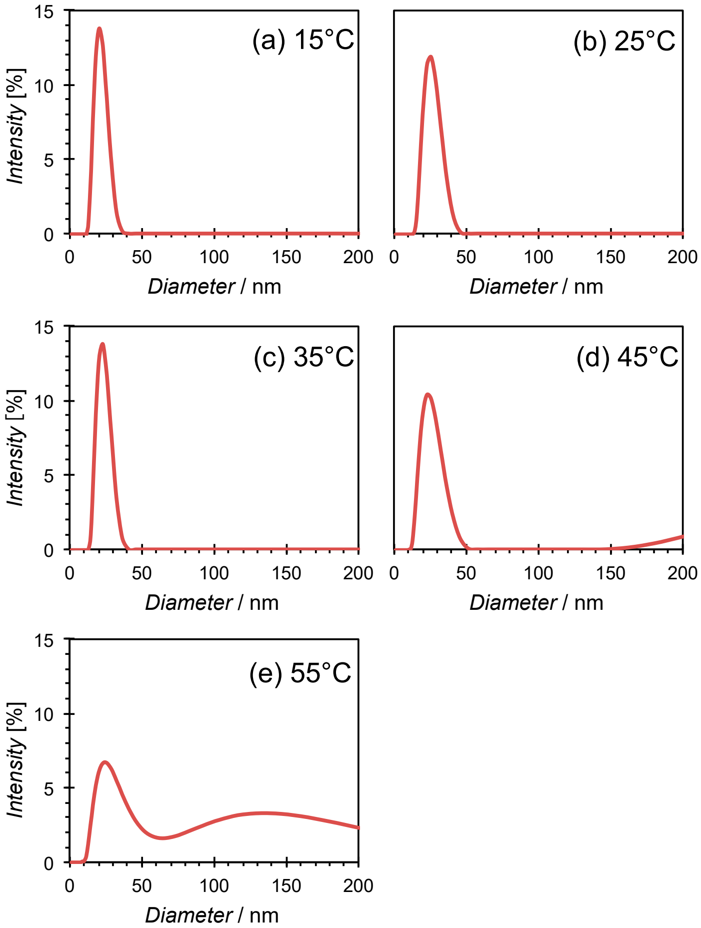

Figure S1. Particle diameter distributions of W_S = 0.005 and X_C = 0.6 at (**a**) 15 °C, (**b**) 25 °C, (**c**) 35 °C, (**d**) 45 °C, (**e**) 55 °C.

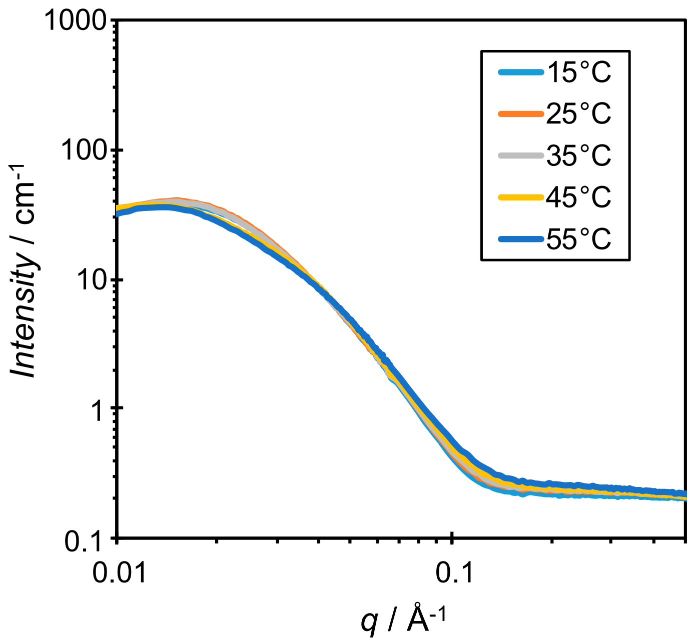

Figure S2. SANS results at different temperatures for dispersions of the SL-$ChEO_{10}$ system (X_C = 0.6, W_S = 0.02).

Table S1. SANS fitting parameter.

**Back Ground** b	**0.224 cm**$^{-1}$
Scale s	1.1255 [-]
Length L	34.8 nm
Face thickness T_f	0.8 nm
Rim thickness T_r	1.0 nm
Core radius R	2.2 nm
Face SLD d_f	0.5262 × 10^{-6} Å^{-2}
Rim SLD d_r	0.6510 × 10^{-6} Å^{-2}
Core SLD d_c	−0.3694 × 10^{-6} Å^{-2}
Solvent SLD d_{solv}	3.127 × 10^{-6} Å^{-2}
Charge Z	11.441 e
Dielectric constant ε	75.837 [-]
Volume fraction φ	0.0361 [-]

Note: SLD stands for scattering length density.

© 2020 by the authors. Licensee MDPI, Basel, Switzerland. This article is an open access article distributed under the terms and conditions of the Creative Commons Attribution (CC BY) license (http://creativecommons.org/licenses/by/4.0/).
